# Salt intake was higher among males and those with high BMI and waist circumference: introduction to the Malaysian Community Salt Survey (MyCoSS), a population-based salt intake survey in Malaysia

**DOI:** 10.1186/s41043-021-00229-y

**Published:** 2021-05-31

**Authors:** Rashidah Ambak, Feng J He, Fatimah Othman, Viola Michael, Muhammad Fadhli Mohd Yusoff, Tahir Aris

**Affiliations:** 1 Institute for Public Health, National Institutes of Health, Ministry of Health Malaysia, Selangor, Malaysia; 2 Wolfson Institute of Preventive Medicine, Barts and The London School of Medicine & Dentistry, Queen Mary University of London, London, UK; 3 Disease Control Division, Ministry of Health Malaysia, Putrajaya, Malaysia

**Keywords:** Urinary sodium, Salt intake, Malaysia, MyCoSS

## Abstract

**Background:**

Recognising that excessive dietary salt intake is associated with high blood pressure and adverse cardiovascular health, the Ministry of Health Malaysia conducted the Malaysian Community Salt Survey (MyCoSS) among Malaysian adults. This paper introduced MyCoSS projects and presented findings on the salt intake of the Malaysian adult population.

**Methods:**

MyCoSS was a nationally representative survey, designed to provide valuable data on dietary salt intake, sources of salt in the diet, and knowledge, perception, and practice about salt among Malaysian adults. It was a cross-sectional household survey, covering Malaysian citizens of 18 years old and above. Multi-stage-stratified sampling was used to warrant national representativeness. Sample size was calculated on all objectives studied, and the biggest sample size was derived from the knowledge on the effect of high salt on health (1300 participants). Salt intake was estimated using a single 24-h urine collection and its sources from a food frequency questionnaire. Knowledge, attitude, and practice were determined from a pre-tested questionnaire. All questionnaires were fully administered by trained interviewers using mobile devices. Anthropometric indices (weight, height, and waist circumference) and blood pressure were measured using a standardised protocol. Ethical approvals were obtained from the Medical Research Ethics Committee, Ministry of Health Malaysia, and Queen Mary University of London prior to conducting the survey.

**Results:**

Findings showed that the average sodium intake of Malaysian adults (3167 mg/day) was higher than the WHO recommendation of 2000 mg/day. Daily intake was significantly higher among males and individuals with higher BMI and higher waist circumference.

**Conclusion:**

Salt intake in the Malaysian population was higher than the WHO recommendation. MyCoSS’s findings will be used for the development and implementation of national salt reduction policy. A successful implementation of a national salt reduction programme in Malaysia will benefit the whole population.

## Background

Cardiovascular diseases (CVD), including strokes, heart attacks, and heart failure, are the foremost cause of death in Malaysia and worldwide. Hypertension contributed to incidence of strokes (62%) and coronary heart disease (49%) [1]. There is irrefutable evidence that high salt intake is the major cause of hypertension. Reducing salt intake to the World Health Organization (WHO) recommended level of less than 5 g/day could prevent about 1.65 million CVD deaths per year globally [2]. A paper in the Lancet demonstrated that a 15% reduction in population salt intake could avert 8.5 million cardiovascular deaths over 10 years in 23 developing countries and result in major cost savings to individuals, their families, and the health services [3]. Indeed, a modest reduction in salt intake is more, or at the very least just, as cost-effective as tobacco control in terms of reducing CVD, the leading cause of death and disability in Malaysia and worldwide [4]. Salt reduction has shown other benefits on health [1], namely, reduced risk of stomach cancer [5], kidney disease, and osteoporosis [1], and lower risk of obesity [6] directly or indirectly through a reduction in soft drink consumption [7].

Salt intake has been included as an indicator under the Non-Communicable Disease Global Monitoring Framework [8] adopted at the World Health Assembly in 2014, with reporting requirements to the United Nations General Assembly in New York every 5 years, starting in 2015. Malaysia started a salt reduction initiative in 2010, and the Ministry of Health announced to reduce salt in 11 food items, e.g. soy sauce. A committee was set up under the Non-Communicable Disease (NCD) Section, Disease Control Division, Ministry of Health, in collaboration with several agencies to raise salt awareness and promote salt reduction in Malaysia.

The salt reduction initiative in Malaysia is timely, particularly in view of the rising trend of hypertension. The prevalence of hypertension in adults of over 18 years has increased from 21% in 1996 to 33% in 2011 [9]. This figure continues to rise. It is estimated that 7.6 million adults will have hypertension by 2020 [10]. Prior to MyCoSS, there was no national database on salt intake. A small study in 84 young adults (age 19–30 years) showed that average salt intake, measured by 24-h urinary (24HU) sodium, was 9.2 g/day in 2009 [11]. In 2012, a study was carried out in 445 individuals employed by the Ministry of Health (MOH) Malaysia which showed that average salt intake was 8.4 g/day, with over 70% of the participants having salt intake above the WHO recommended level of 5 g/day [12]. A more recent study in 2015 was conducted among 1116 individuals employed by MOH with 24-h urine (24HU) collection, 24-h diet recall, food frequency questionnaire (FFQ), and knowledge, attitude and practice (KAP) questionnaire [13]. The results showed that mean salt intake, measured using 24 HU sodium excretion, was 7.2 g/day. The main sources of dietary salt intake were soy sauce, fried rice, omelette, *nasi lemak* (Malay cuisine dish of fragrant rice cooked in coconut milk and pandan leaf, and served with a hot spicy sauce, fried anchovies, hard-boiled or fried egg, cucumber slices, and roasted peanuts), and *roti canai* (flat bread). By food groups, cooked grains and sauces were the primary contributors to dietary salt intake. Regarding knowledge, attitude, and practice, most of the participants had knowledge on serious health problems related to high salt intake, especially high blood pressure. More than half of the participants responded that lowering sodium intake was very important to them. The participants frequently controlled their salt intake, and most of them never added salt to the food at the dining table, but often or always used salt in cooking.

The studies on health workers and others using small samples cannot be generalised to the wider population [13]. Hence, the Malaysian Community Salt Survey (MyCoSS) was conducted to determine the level of salt intake, main sources of salt in the diet, and knowledge, attitude, and practice (KAP) in a nationally representative sample of the Malaysian adult population. Our study will provide key data which are urgently required for effective implementation of Malaysia’s salt reduction programme. This paper introduced MyCoSS project and presented findings on the salt intake. This journal supplement also presented findings on other scopes of the study: sodium sources, KAP, anthropometric measurements, hypertension, potassium intake, and having meals away from home, and their associated factors. A sub-study on development of sodium equation to estimate sodium intake among the Malaysian adults was also included in this supplement.

## Methods

### Study design and sampling

MyCoSS was a nationally representative household survey. The target population was participants residing in non-institutional living quarters. Institutional residents (those staying in hospitals, hotels, hostels, prisons etc.) were excluded from the survey. The sample size was calculated using a formula for estimating population prevalence. Sampling was designed to cover both urban and rural areas for every state in Malaysia. Calculations were done on all objectives, and the biggest sample size was derived from the knowledge on the effect of high salt on health, with an estimated sample size of 1300 participants. To ensure national representativeness, this cross-sectional study applied a stratified cluster sampling method. Sampling was designed to cover both urban and rural areas for every state in Malaysia. Residential units were randomly selected by the Department of Statistics Malaysia, and one respondent was selected from a household. Sample size for each state was calculated proportionally to the state’s population size.

### Participant characteristics

Malaysian citizens aged 18 years and above were included. Those who were pregnant; recently began diuretic therapy (<4 weeks); having menses; diagnosed to have kidney disease, heart failure, or liver disease; and with difficulty of urine collection were excluded from the study. Participants who agreed to participate in this study and had met the eligibility criteria were provided an information sheet that explained the purpose and detailed information of the study. They were asked to sign a consent form which included allowing IPH researchers to keep their urine sample for 10 years for future research.

### Data collection and study instruments

**Fig. 1:**
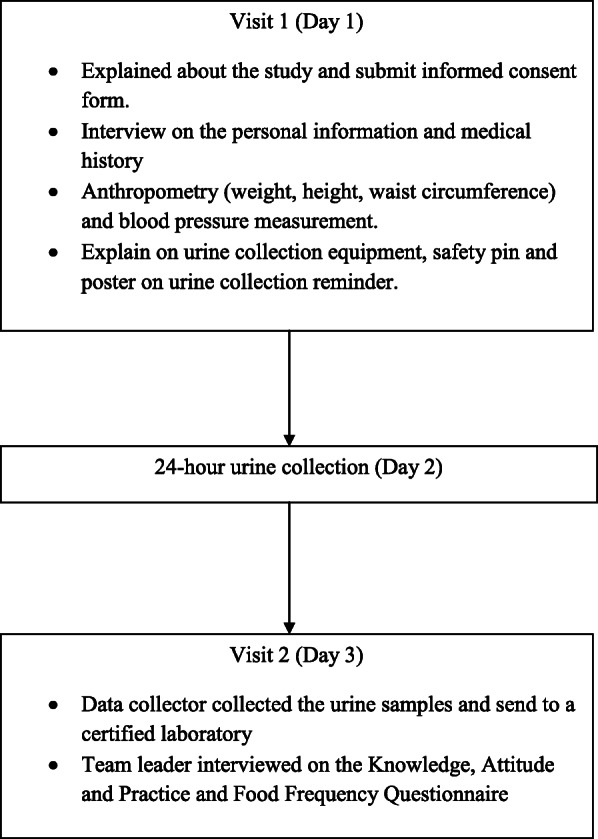
Data collection flowchart

Data collection started from October 2017 and completed in March 2018. There were two separate visits arranged for each respondent (Fig. 1). Data collections via face-to-face interviews were carried out at the respondent’s home using mobile tablets based on the system developed for this study. Questionnaires were pre-tested in the field, modified, and finalised for full survey implementation. Data was stored, backed up in the secure digital card, sent to the institute’s server, and saved in the dataset folders according to the downloaded completion time.

The study questionnaires consisted of personal information; medical and health history; physical activity; knowledge, attitude, and practice (KAP); and food frequency questionnaire (FFQ) (Table 1).

**Table 1:** Component and parameters studied in MyCoSS

Component	Parameters studied
Personal information	Date of interview, name, address, phone number, identity card number, gender, age, occupation, ethnicity, marital status, academic qualification, monthly individual income and monthly household income.
Medical and health history	Ever diagnosed for heart disease, stroke, diabetes, hypertension and hypercholesterolemia and kidney disease. Consumption of diuretics and any special diet (diabetic diet, low-calorie or low-salt diet).
Anthropometry and blood pressure measurement	Weight (TANITA Digital Weighing Scale HD 319-TANITA Corp., Tokyo, Japan), height (SECA Portable Stadiometer 213-SECA GmbH & Co. KG, Hamburg, Germany), waist circumference (SECA Bodymeter 206, Germany) and blood pressure (Omron HBP-1300-Omron Corporation, Kyoto, Japan).
Knowledge, attitude and practice, and dietary behaviour	Knowledge on effect of salt on health, importance of lowering salt, perception of how much salt consumed, salt/sauce/enhancer usage during cooking and at the table, and actions taken to lower salt intake [14]. Dietary pattern on intake of breakfast, lunch and dinner (frequency in a week and source of food).
Dietary intake (high sodium food sources)	Food frequency questionnaire [15].
24-h urine collection	Urine volume, sodium and creatinine measurement

The team leader interviewed the participants using a pre-tested and validated FFQ, which was adapted from a previous salt study in Malaysia [13]. Responses were recorded on daily or weekly or monthly frequency, as well as portions consumed. The knowledge, attitude, and practice (KAP) questionnaire was adapted from the World Health Organization/Pan American Health Organization protocol for population level sodium determination [15]. All anthropometric measurements and blood pressure (weight, height, waist circumference) were done using validated and calibrated instruments. Body mass index (BMI) was calculated as the ratio of weight in kilogrammes to the square of height in metre (kg/m^2^) and categorised based on the WHO 1998 guidelines of underweight (<18.5 kg/m^2^), normal-weight (18.5–24.9 kg/m^2^), pre-obese (25.0–29.9 kg/m^2^), and obese (≥ 30.0 kg/m^2^) [14]. High waist circumference was categorised as >90 cm for men and >80 cm for women (WHO/IASO/IOTF) [14, 16]. Blood pressure was measured using a digital blood pressure machine Omron HBP-1300 and referred to the Ministry of Health Clinical Practice Guideline [17]. Detail methodology of the FFQ and KAP are highlighted in other articles in this supplement: S02 High sodium food consumption pattern among Malaysian population [18] and S03 Knowledge, Attitude and Behaviour on Salt Intake and its Association with Hypertension in Malaysian Population: Findings from the MyCoSS (Malaysian Community Salt Survey) [19].

### Twenty-four-hour urine collection

The participants were given oral and printed instructions on the technique to accurately collect a single 24 HU. Each participant was provided with a 5-L screw-capped plastic collection bottle, a urine collection jug, plastic carrier bags, a safety pin for attaching to underclothes, and a poster (as a reminder for urine collection). The team leader made a phone call to remind participants of the urine collection. The participants discarded the first urine of the day in the morning and collected all urine for the following 24 h. The last urine collection was determined as the first urination on the second day of collection. The participants recorded the urine collection time and notified any missed collection to the data collectors. Sodium content in the urine was measured using indirect ion-selective electrode method, while urinary creatinine was measured using the Kinetic Jaffe method (alkaline picrate with Lloyd’s reagent) using the Architect C machine in a laboratory. Incomplete 24 HU sample was determined as urinary creatinine <4 mmol/day for women or <6 mmol/day for men, 24 HU volume of <500 ml for both sexes [20]. Conversion from mmol to grams of salt was made by dividing by a factor of 17, and the conversion from sodium (Na) to salt (NaCl) was by multiplying by a factor of 2.542 [20]. Sodium intake was compared to Malaysia’s sodium intake recommendation of less than 2000 mg/day.

### Statistical analysis

This article focused on findings on the salt intake of the Malaysian adult population. Determination of salt intake was done using complex sample design analysis. Prevalence estimates and 95% confidence intervals of the studied variables were computed. The data was analysed using the complex sample functions in IBM SPSS version 20 and Stata version 14.

## Results

### Salt intake of the Malaysian adults

A total of 1047 participants were interviewed, 960 of whom provided urine samples. Based on the criteria for assessing the completeness of 24-h urine collection, 798 urine samples were complete and included in the analysis (76.2% inclusion). The participants aged between 18 and 85 years old with a mean age of 49 years (95% CI 47, 51). Mean body weight was 67.8 kg (95% CI 66.4, 69.3), and mean BMI was 26.6 kg/m^2^ (95% CI 26.0, 27.1). Mean waist circumference for male was 91.1 cm (95% CI 89.4, 92.9) and female 88.6 cm (95% CI 86.6, 90.5). Total 24-h urine volume was between 500 and 5780 ml.

Table 2 presents the mean 24-h sodium intake level (mmol/day) by the characteristics of the participants. The mean 24 HU sodium intake was 3167 mg/day (95% CI 2987, 3346), which corresponds to 138 mmol/day or 7.9 g of salt or 1.6 tsp of salt. About 79% of the participants consumed high sodium (more than 2000 mg sodium per day). Sodium intake was significantly higher among males (3519 mg/day) compared to females (2790 mg/day) (*t*=4.21, *p*<0.001). Sodium intake was the highest among those aged 25–34 and 35–44 years and lowest among the elders (more than 65 years). Highest sodium intake was seen among never-married participants. Those working in the public sector, private sector, and self-employed showed higher sodium intake compared to other job categories. Overall those with higher BMI consumed significantly higher sodium compared to participants with lower BMI (*F*=18.19, *p*<0.001). Male and female with normal waist circumference showed a significantly lower sodium intake compared with high waist circumference (t=5.11, *p*<0.001 and t=4.86, *p*=0.001 respectively). Nevertheless, their sodium intake exceeded the recommended intake (>2000 mg/day).

**Table 2:** Study characteristics and mean sodium intake (mg/day) analysed using 24-h urinary excretion

Characteristics	Unweighted count	%	Population size	Mean	95% confidence interval	
					Lower	Upper
**Malaysia**	798	100	14,243,927	3167	2987	3346
**Gender**						
Male	340	51.7	7,365,184	3519	3209	3828
Female	458	48.3	6,887,075	2790	2650	2931
**Age group (years)**						
18–24	53	6.6	955,086	3266	2748	3783
25–34	117	14.7	1,983,140	3556	3099	4013
35–44	130	16.3	2,447,950	3531	3083	3980
45–54	175	21.9	3,160,650	2955	2751	3159
55–64	190	23.8	3,232,280	3047	2790	3305
65+	133	16.7	2,473,153	2882	2507	3257
**Location**						
Urban	319	40.0	10,809,259	3254	3024	3483
Rural	479	60.0	3,443,000	2894	2746	3041
**Ethnicity**						
Malay	501	62.8	9,480,360	3214	2992	3436
Chinese	87	11.0	1,681,915	2926	2575	3278
Indian	44	5.5	1,205,438	3408	2399	4417
Bumiputera Sabah	89	11.1	1,352,484	2879	2489	3270
Bumiputera Sarawak	64	8.0	342,464	2992	2740	3244
Others	13	1.6	189,599	3772	2928	4616
**Marital status**						
Never married	94	11.8	1,644,183	3407	3060	3754
Married	594	74.4	11,056,754	3224	3018	3430
Separated	24	3.0	351,593	2374	1812	2937
Widow/er	86	10.8	1,183,117	2528	2223	2833
**Household income (RM)**						
< 1000	239	29.9	3,357,918	2943	2749	3137
1000–1999	153	19.2	2,516,305	3262	2901	3623
2000–2999	131	16.4	2,284,080	3162	2700	3624
3000–3999	90	11.3	1,707,701	2963	2487	3439
> 4000	185	23.2	4,386,255	3365	3049	3682
**Academic level**						
None	64	5.1	731,726	2729	2112	3346
Primary education	167	18.2	2,587,785	3051	2667	3436
Secondary education	383	51.1	7,286,332	3232	2987	3476
Tertiary education	184	25.6	3,646,416	3207	2956	3457
**Occupation group**						
Public sector	117	14.7	1,988,275	3326	2872	3780
Private sector	126	15.8	2,451,061	3338	2990	3686
Self-employed	179	22.4	3,278,506	3469	3038	3900
Housewives	214	26.7	3,364,461	2905	2722	3088
Unemployed	113	14.2	1,943,337	2887	2537	3237
Student	15	1.9	280,201	2919	2183	3655
Others	34	4.3	946,419	2919	2426	3411
**Body mass index**^**a**^ **(kg/m**^**2**^**)**						
Underweight (<18.5)	35	4.5	640115	2029	1698	2361
Normal (18.5–24.9)	285	35.2	5009039	2877	2671	3082
Pre-obese (25.0–29.9)	287	37.5	5350422	3285	3007	3562
Obese (≥ 30.0)	191	22.8	3252684	3643	3216	4070
**Waist circumference (male)** ^**b**^**(cm)**						
< 90	410	48.7	6,945,737	2811	2639	2982
≥ 90	387	51.3	7,311,322	3506	3233	3777
**Waist circumference**^**b**^ **(female)** ^**b**^ **(cm)**						
< 80	180	20.1	2,866,271	2675	2427	2923
≥ 80	620	79.9	11,396,476	3291	3098	3483
**Blood pressure**^**c**^ **(mmHg)**						
< 140/90	489	61.3	8,986,647	3180	2952	3408
≥ 140/90	309	38.7	5,265,613	3144	2927	3360

^a^BMI categories referring to WHO (1998) [14]

^b^Waist circumference referring to WHO/IASO/IOTF (2000) [16]

^c^Blood pressure referring to the Malaysian Ministry of Health Guideline [17]

## Discussion

The mean sodium intake reported in our study was lower than that estimated in other smaller studies in Malaysia: 3412 mg/day (19- to 30-year-old university students, *n*=84) [11], 3425 mg/day (18- to 59-year-old health staffs in 2012, *n*=445) [12]. The present study reported a lower intake of sodium as it included elderly participants aged more than 60 years old. The elderly consumed less sodium due to lower intake of food as they aged and medical restrictions [21]. This resulted in a lower population sodium intake. Malaysia’s sodium intake was also lower than reported by Singapore’s National Nutrition Survey 2018 (9.0 g/day) [22]. This is probably due to Malaysia’s main source of sodium consumption were from fried vegetables (adding salt during cooking), bread, and soy sauce (as highlighted in the article by Ahmad et al. in this supplement) [18], compared to Singapore’s sodium main consumption from seasonings, salt, and sauces used in food preparation [22]. Malaysia’s sodium intake was also lower than the adult (aged 19 to 64 years) UK population, 148 mmol/day [23]. Higher sodium consumption in the UK (8.4 g/day) was contributed from sodium added in the production processes (61%), whilst sodium added during cooking or eating contributed only 18% of the daily sodium intake. Cereals (and products) and meat (and products) contributed to the biggest sodium intake in the UK [24]. This study reported an almost similar sodium intake with a study conducted among health staff aged 18 to 59 years old in 2015 (2989 mg/day, *n*=1116) [13].

Overall, sodium intake of Malaysian males (153 mmol/day) and females (122 mmol/day) was lower compared to other countries such as the UK (males, 161 mmol/day; females, 127 mmol/day) [25], the USA (males, 180–190 mmol/day; females, 130–150 mmol/day) [25], China (males, 252 mmol/day; females 234 mmol/day) [26], and Singapore (males, 175 mmol/day; females 122 mmol/day) [22]. In addition, sodium intake was significantly higher among Malaysian males compared to females. These findings are consistent with most studies in other countries such as the USA [27], Spain [28], Sweden [29], and China [26]. This is possibly due to dietary behaviour differences of consuming food outside of home [30–32]. Moreover, male’s higher food intake requirement leads to higher sodium consumption [33].

By age, sodium intake was the highest among those aged 25–34 and 35–44 years, while the elders (more than 65 years) reported the lowest sodium intake. This echoes findings from Singapore whereby the highest sodium intake was reported among those aged 30–49 years age group and was lower for older age groups [22]. This might be due to decreasing food intake among older people, and consequently, lower sodium ingestion. The UK and Spanish population also showed a decreasing trend of sodium intake starting from a later age of 50 years [23, 28].

The current study confirmed that sodium intake was significantly increased among adults with higher BMI and waist circumference. This could be explained by consumption of a larger amount of food and energy-dense food that is accompanied by more sodium in the daily diet [18]. Other studies have also observed high sodium intake among those with higher BMI and waist circumference [33, 34].

Findings showed no significant differences in sodium intake by location. Due to logistic problems in sending urine samples to laboratories and limited allocation, participants in secluded areas were not included in this study. Those studied still have access to ready-to-eat and local fast foods (which are high in calories, sugar, and salt) sold at street stalls, hawker centres, night markets, and convenient stores. This might explain why there was no difference in sodium intake by location in Malaysia [35]. The Malaysian Adult Nutrition Survey 2003 also reported no differences in sodium intake by location [36].

Malaysia is a multiracial nation with the Malay, Chinese, and Indian as major ethnics. Hence, the cuisines are multicultural in nature. Cuisines from the Malays, Chinese, and Indians are available almost everywhere in Malaysia and eaten by all ethnics in Malaysia. This might explain the insignificant result related to ethnicity. Further, there was an imbalance participant numbers according to ethnicity ratio, causing direct comparison to be difficult. Larger numbers of participants from other ethnicities are needed to represent the Malaysian population.

The strength of the study is its representativeness population and 24-h urine collection to determine population salt intake. A stringent protocol was deployed to minimise risk of incomplete collection of 24-h urine. The respondents were provided with instructions on urine collection and reported their start and end of urine collection. Further, the completeness of the urine was determined using the 24HU creatinine measurement and by the volume collected. MyCoSS carried out a sampling frame which is stratified by urban/rural living quarters and by states. Hence, this survey is considered to be nationally representative. However, this study has some limitations, e.g. para-aminobenzoic acid (PABA) tablet was not used to verify the completeness of the 24 HU. In spite of the stringent protocol, we cannot rule out incomplete collection of 24-h urine samples as it is very common and occurred in almost all population studies. Furthermore, for the assessment of salt intake, we did not include non-urine loss of sodium which accounts for approximately 10% of total salt intake. As such, the salt intake reported in our study represents an underestimated level. Despite this, 79% of the population has salt intake above the WHO recommended maximum. Urgent action is therefore needed to reduce salt intake across the population. In addition, secluded geographical areas had to be excluded from the sampling frame due to logistic reasons and budget limitation. Another limitation is that we collected a single 24-h sample despite repeated measurements of two or more urine samples are preferred to estimate day-to-day variations in diet intake and habitual intake.

## Conclusion

The salt intake in Malaysian adults is higher than the WHO recommendation. Intake is higher among males, with high BMI and waist circumference. The information generated from this study is fundamental for strengthening the implementation of the national salt reduction programme in Malaysia. They will serve as baseline information in monitoring the trend of salt intake over time and in evaluating the effectiveness and cost-effectiveness of the country’s salt reduction programme. The results will be useful to inform policy makers, programme managers, health professionals, the food industry, non-governmental organisations and other stakeholders to make concerted efforts to reduce salt intake in Malaysian population. The findings will also provide an important data source to raise salt awareness among the general public, resulting in a successful public health campaign to reduce salt intake in Malaysia.

## Data Availability

The datasets used and/or analysed during the current study are available from the corresponding author on reasonable requests.

## Abbreviations

MyCoSS: Malaysian Community Salt Survey
MREC: Medical Research Ethics Committee
CVD: Cardiovascular disease
WHO: World Health Organization
FFQ: Food frequency questionnaire
KAP: Knowledge, attitude and practice
